# Case Report: Two instances of lung metastasis in low-grade endometrial stromal sarcoma

**DOI:** 10.3389/fonc.2025.1588783

**Published:** 2025-07-25

**Authors:** Anying Long, Xiaoxue Tian, Yao Li, Shuai Luo, Jinjing Wang

**Affiliations:** Department of Pathology, Affiliated Hospital of Zunyi Medical University, Zunyi, Guizhou, China

**Keywords:** endometrial stromal sarcoma, metastasis, lung, pathology, diagnosis

## Abstract

**Background:**

Low-grade endometrial stromal sarcoma (LGESS) with pulmonary metastasis is a rare malignant mesenchymal tumor. LGESS is composed of cells resembling proliferative endometrial stromal cells. It grows slowly and has a favorable prognosis, but late - term recurrence and metastasis are common. Thus, long - term regular follow - up is needed, and the possibility of tumor recurrence or metastasis should be considered when lesions appear later.

**Case demonstration:**

Case 1: A 55-year-old woman underwent a total hysterectomy 11 years ago for adenomyosis. She was later diagnosed with low-grade malignant uterine stromal sarcoma. She was admitted to the hospital due to “discovery of a pulmonary nodule 5 days ago.” Chest CT showed a nodule in the outer basal segment of the left lower lobe of the lung. Subsequently, she underwent “a single-port thoracoscopic wedge resection of the left lower lung lobe and closed thoracic drainage.”The postoperative pathology confirmed a low-grade endometrial stromal sarcoma with lung mety -30astasis. At the initial staging, no lung metastasis was detected. After surgery, she underwent EBRT radiotherapy. During the 12 - month follow - up, no recurrence was observed. Case 2: A 46-year-old woman underwent a total hysterectomy for a uterine mass at an external hospital two months ago. The postoperative pathology diagnosed her with low-grade endometrial stromal sarcoma. She was admitted to the hospital due to “chest pain for two months.” Chest CT indicated a nodule near the pleura of the left lower lobe of the lung. Subsequently, “a biopsy of the left pleural nodule was performed,” and the postoperative pathology confirmed metastatic endometrial stromal sarcoma to the left pleura. At the initial staging, no lung metastasis was detected. After surgery, she underwent EBRT radiotherapy. During the 4 - year follow - up, no recurrence was observed.

**Conclusions:**

Low-grade endometrial stromal sarcoma (LESS) lung metastasis is common in middle-aged and elderly women. While the overall survival rate is good, patients with long-term recurrence or metastasis, especially those with localized or non-metastatic tumors, face a high risk of disease progression. Currently, there is no standardized chemotherapy regimen for this condition. We report two cases of LGESS pulmonary metastasis, analyzing clinical features, histological morphology, immunohistochemistry, and differential diagnosis to enhance understanding of this condition. Without a medical history, it is easy to misdiagnose, particularly in cases of LGESS, where atypical symptoms can lead to misdiagnosis. Regular follow-up, prompt diagnosis, and treatment are crucial for improving prognosis and survival.

## Background

Endometrial stromal sarcoma (ESS) is rare, accounting for 0.2% of primary malignant uterine tumors and 15% of uterine sarcomas (1, 2). Pulmonary metastasis of ESS is particularly uncommon. About 50% of patients with pulmonary metastasis of ESS have no obvious clinical symptoms. Imaging usually shows multiple pulmonary nodules, but may also present as a mass or solitary nodule (3). In the imaging diagnosis of low-grade endometrial stromal sarcoma, pelvic MRI is the preferred method for assessing myometrial invasion and heterogeneous enhancement. Ultrasound is used for preliminary screening, while CT/PET-CT is employed to evaluate tumor metastasis. Due to its rarity, subtle clinical symptoms, and non-specific imaging features, it is often misdiagnosed as a benign fibroid. The definitive diagnosis relies on pathological examination and immunohistochemistry.

This article reports two cases of pulmonary metastasis of ESS, analyzing clinical features, imaging, cytomorphology, histomorphology, immunohistochemistry, and differential diagnosis to enhance understanding of the disease.

## Case demonstration

**Figure 1:**
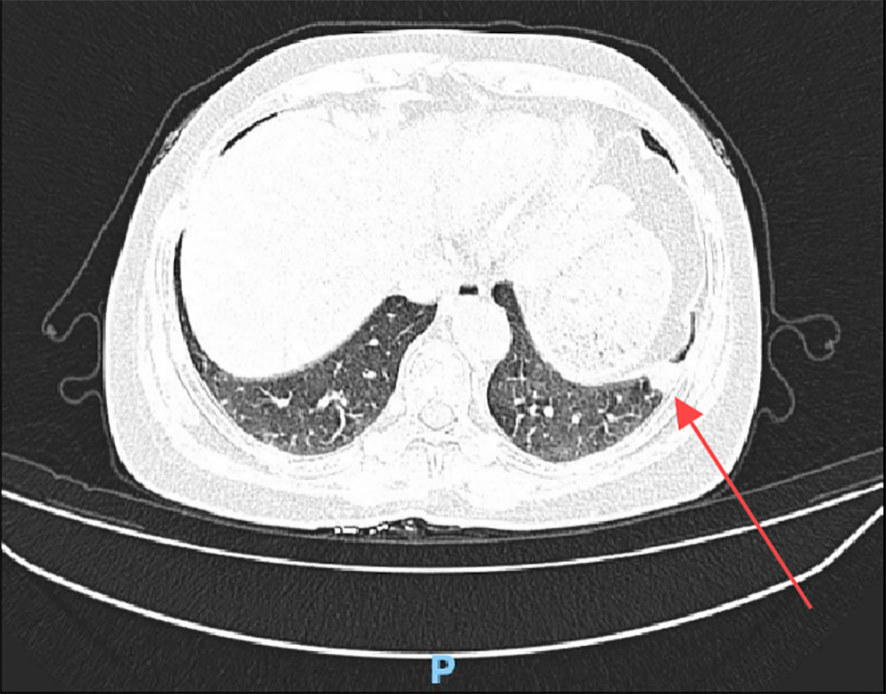
Chest CT shows a high-density nodule in the outer basal segment of the left lower lobe (red arrow).

Case 1:A 55-year-old woman, previously diagnosed with low-grade endometrial stromal sarcoma with vascular invasion after a total hysterectomy 11 years ago, was admitted for a pulmonary nodule detected 5 days prior. Physical examination revealed clear breath sounds with no abnormal rales. Chest CT (**Figure 1**) showed a 15×13mm high-density nodule in the outer basal segment of the left lower lobe, with no significant enhancement on contrast scan, classified as LU-RADS 4A.

In view of the obvious pulmonary nodules and the history of low-grade endometrial stromal sarcoma, the patient underwent “single-chamber thoracoscopic wedge resection of left lower lung + closed thoracic drainage” and was sent for pathological examination.

Gross pathology: Case 1 revealed a 20×16×8mm gray-red nodule in the wedge-shaped lung tissue (120×40×15mm), with unclear margins.

**Figure 2:**
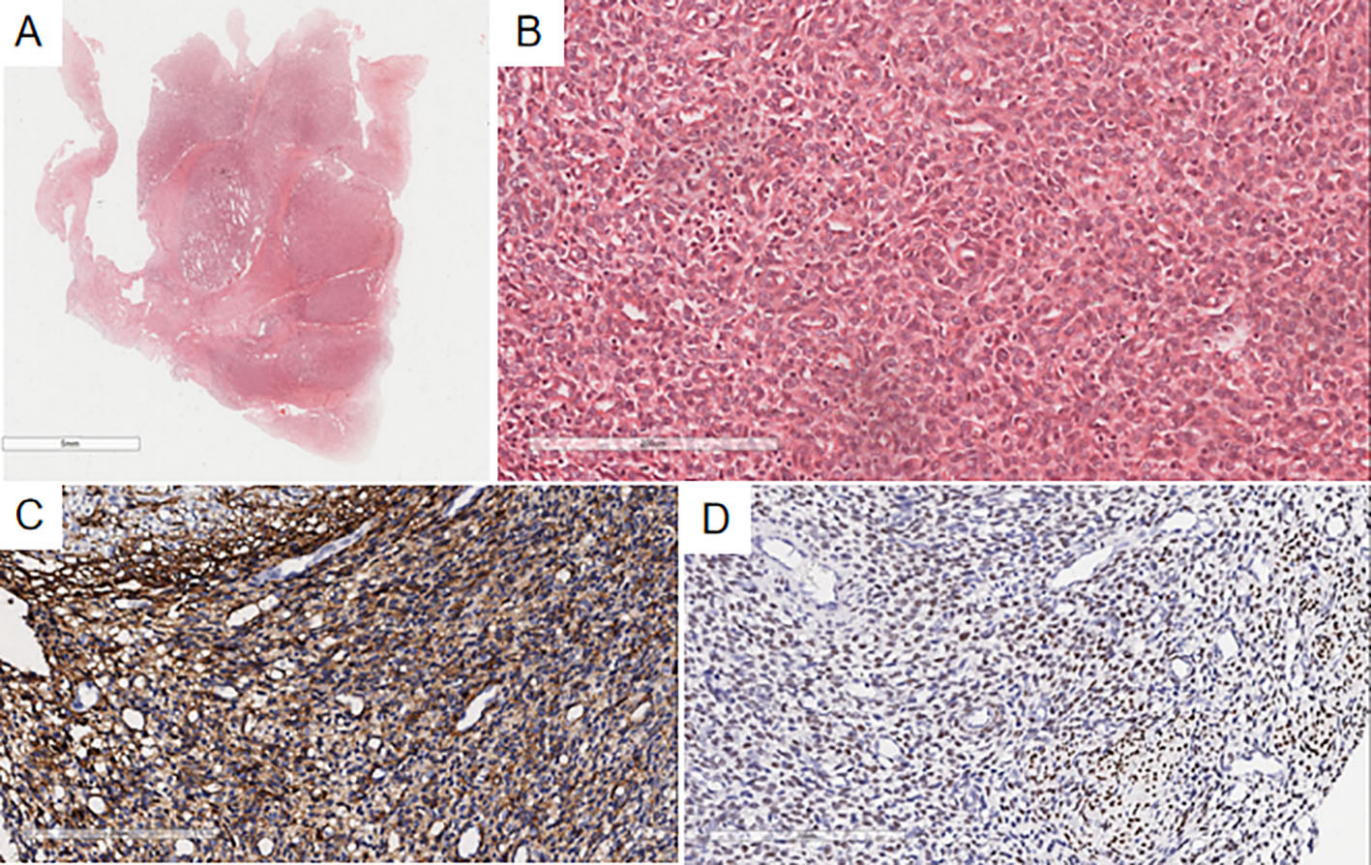
Microscopy shows a well-demarcated nodule in the uterine myometrium with irregular edges **(A)**, infiltrated by numerous short spindle-shaped tumor cells **(B)**. Immunohistochemistry indicates CD10(+) **(C)** and ER(+) **(D)** in the uterine myometrial short spindle-shaped tumor cells.

**Figure 3:**
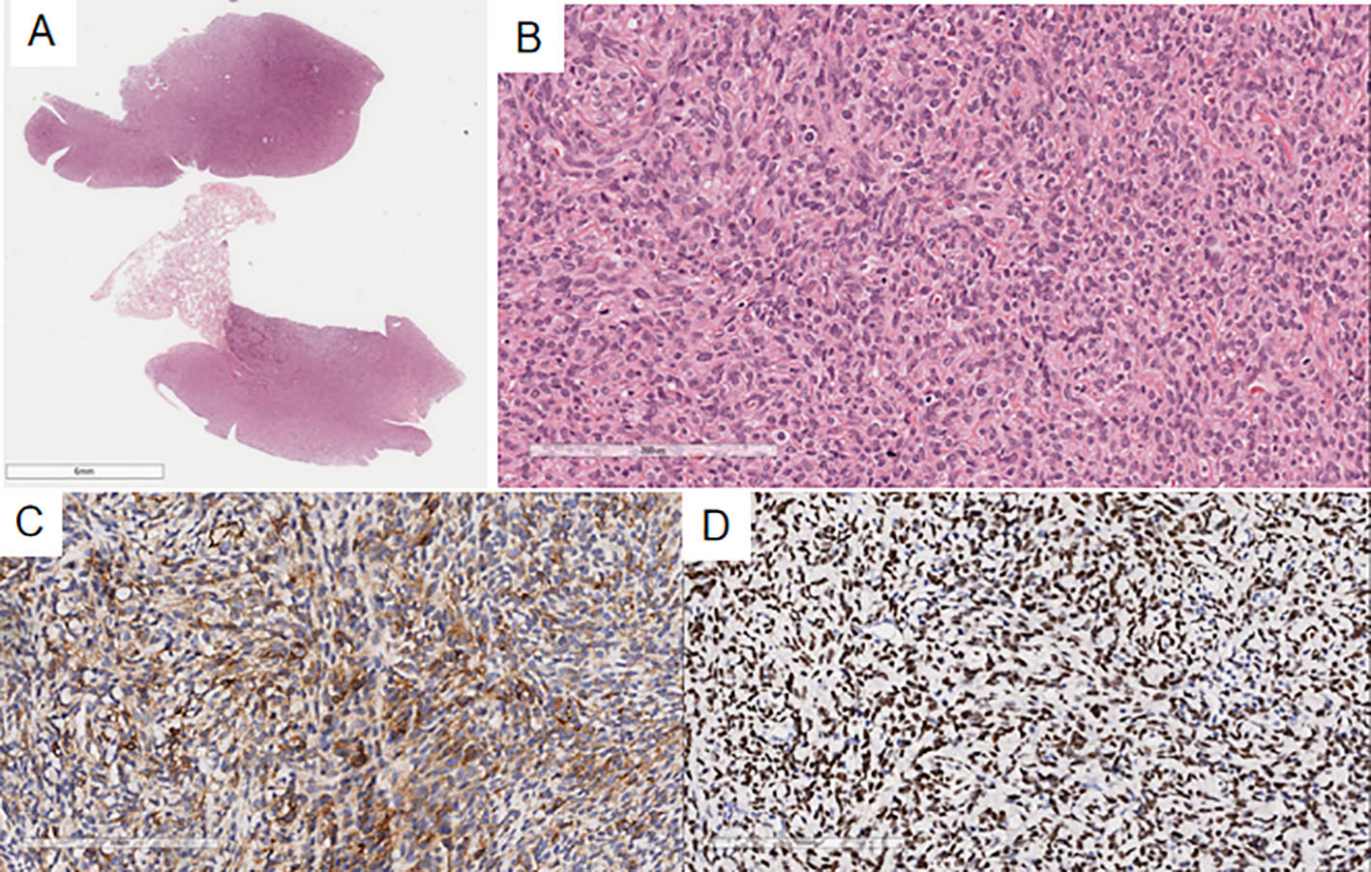
In the pulmonary nodule, the tumor margin is relatively clear on low magnification **(A)**, slightly lobulated, and the tumor cells are oval or spindle-shaped on high magnification **(B)**, resembling proliferative endometrial stromal cells. Immunohistochemistry shows CD10(+) **(C)** and ER(+) **(D)** in the spindle cells of the pulmonary nodule.

Microscopic findings: Case 1’s previous endometrial stromal sarcoma (**Figure 2**) consisted of short spindle cell proliferation with small vessel growth, irregular margins, and vascular invasion. Immunohistochemistry: Vimentin(+), CD10(+), ER(+), cyclinD1(-), CK(-), Desmin(-), SMA(-). In the pulmonary nodule (**Figure 3**), the tumor was nested with clear borders, partly lobulated. Tumor cells were oval or spindle-shaped, with scant cytoplasm, uniform size, mild nuclear atypia, and about 4 mitoses/10HPF, resembling proliferative endometrial stromal cells. The stroma contained foamy cells, with uniform chromatin and rare mitoses. Histomorphology was similar to the previous endometrial stromal sarcoma. Immunohistochemistry of the pulmonary nodule spindle cells: Vimentin(+), CD10(+), ER(+), cyclinD1(-), Desmin(-), CK(-), CK7(-), CK19(-), TTF-1(-), EMA(-), Syn(-), Catenin (membrane+), CD34(-), CK5/6(-), S-100(-), SMA(-), STAT6(-), CD21(-), CD23(-), CD35(-), CD68(-), CgA(-), CT(-). Based on history, histomorphology, and immunohistochemistry, the diagnosis was metastatic endometrial stromal sarcoma in the left lower lung.

Case 2: A 46-year-old woman, diagnosed with low-grade endometrial stromal sarcoma post-total hysterectomy 2 months ago, was admitted for chest pain lasting 2 months. Physical examination showed clear breath sounds with no abnormal rales. Chest CT revealed multiple nodules in the left oblique fissure and subpleural area, with the largest measuring 15×22mm, showing significant enhancement on contrast scan.

In view of the obvious pulmonary nodules and a history of low-grade endometrial stromal sarcoma, the patient underwent cytology examination followed by left pleural nodule biopsy.

**Figure 4:**
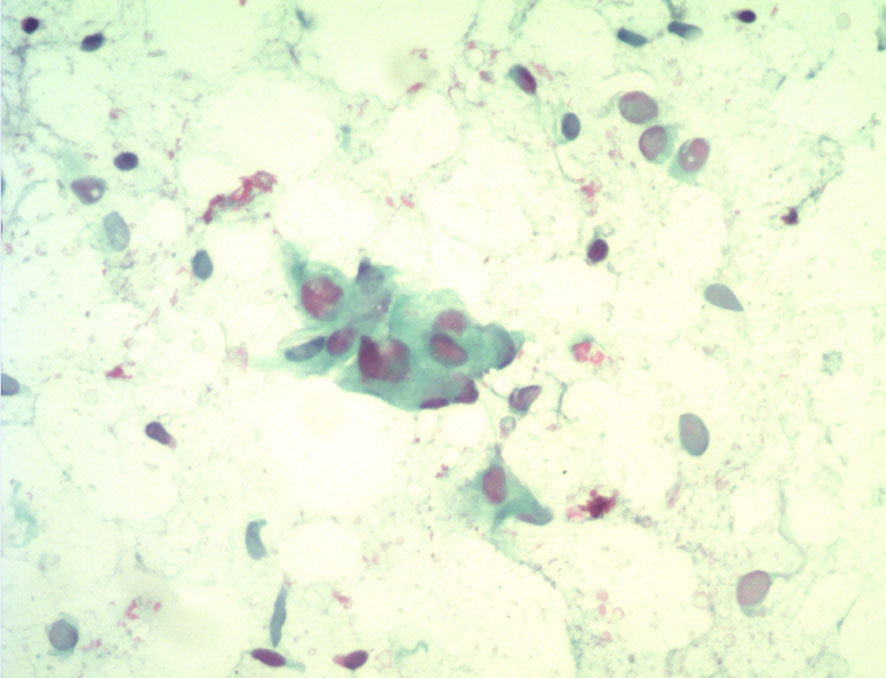
Cytological examination reveals cells arranged in small clusters, with spindle or oval shapes, mild nuclear atypia, and smooth nuclear membranes.

Pathological Findings: Case 2’s cytological examination (**Figure 4**): Cells were arranged in clusters or small groups, with indistinct borders, round or oval shapes, uniform size, mild nuclear atypia, smooth nuclear membranes, pale basophilic cytoplasm, and fine chromatin.

Gross pathology: Case 2 showed a small fragment of broken gray-white tissue (4×3×2mm).

**Figure 5:**
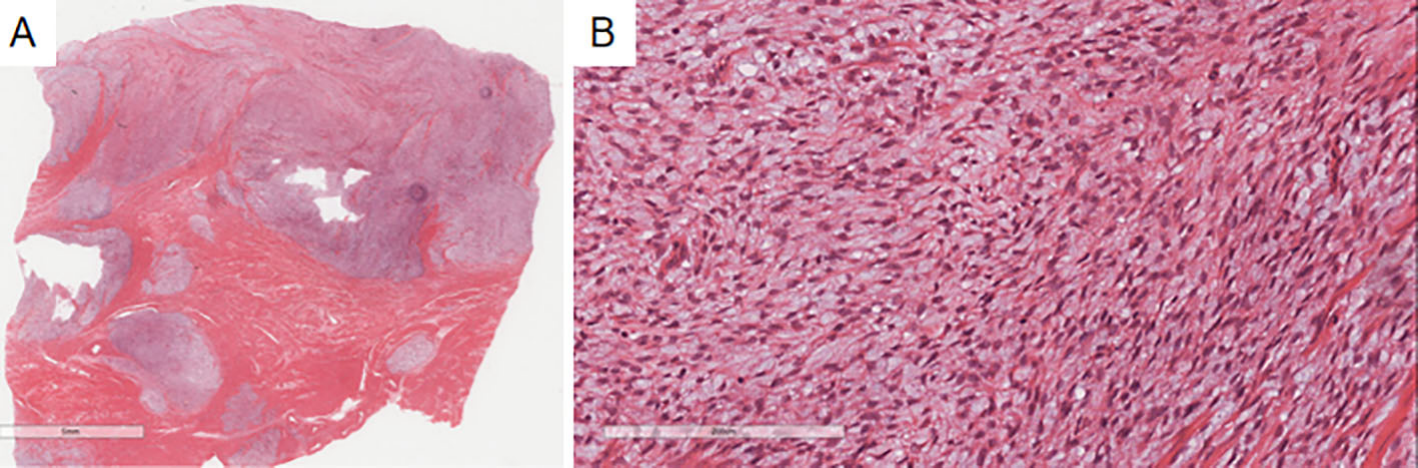
The previous endometrial stromal sarcoma in Case 2 shows a well-demarcated nodule in the uterine myometrium with irregular edges **(A)**, infiltrated by numerous short spindle-shaped cells **(B)**.

**Figure 6:**
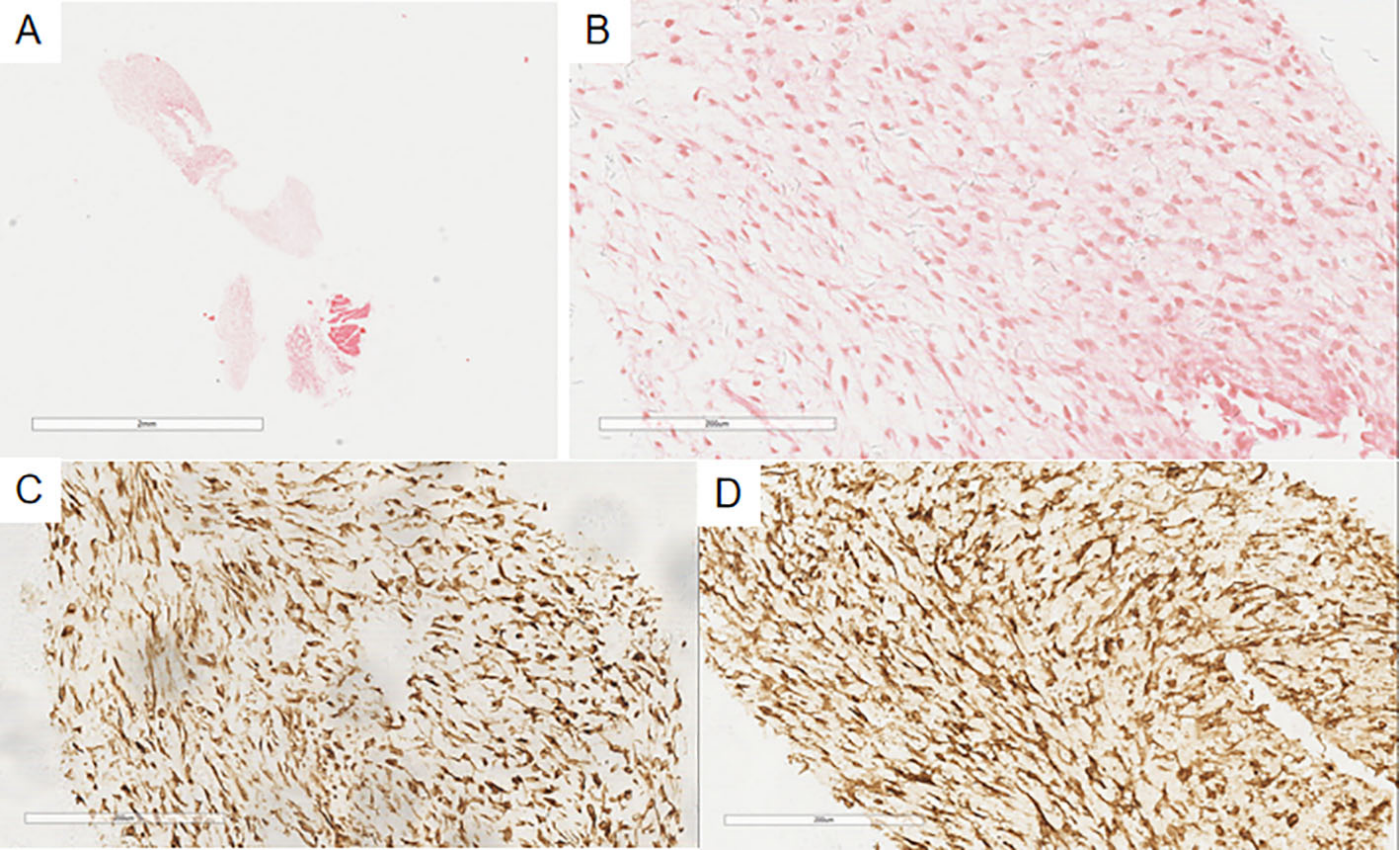
In the pleural puncture tissue of Case 2 **(A)**, there are a few proliferating spindle-shaped tumor cells **(B)**, with myxoid change. Immunohistochemistry shows Vimentin(+) **(C)** and CD10(+) **(D)** in the tumor cells.

Case 2’s microscopic findings: The previous endometrial stromal sarcoma (**Figure 5**) was similar to Case 1. In the pleural biopsy tissue (**Figure 6**), there were few spindle cells with mild atypia, no clear mitoses, and myxoid stroma. Immunohistochemistry: Vimentin(+), CD10(+), CD34(-), S100(-), SMA(-), STAT6(-), CK(-), CR(-), MC(-), Ki-67(20%+), consistent with prior endometrial stromal sarcoma. The diagnosis was metastatic endometrial stromal sarcoma in the left pleura.

After the two patients were diagnosed, both opted for EBRT (External Beam Radiotherapy) as their radiotherapy regimen. In case 1, EBRT was administered to the left lung postoperative area, with a dose of 48Gy over 26 sessions, and aromatase inhibitors were also used. For case 2, EBRT was given to the postoperative area, with a dose of 45Gy over 25 sessions, and aromatase inhibitors were also used. Both patients received radiotherapy after surgery due to concerns about positive margins or residual lesions. Follow-up visits were conducted one year and four years later, respectively, with no recurrence observed. Follow-up for LGESS primarily involved imaging examinations (pelvic ultrasound, MRI, CT, PET-CT) combined with clinical symptoms such as abnormal vaginal bleeding or discharge, abdominal distension, and abdominal pain.

## Discussion

ESS, originating from endometrial stromal cells, is a rare mesenchymal malignancy in genital tract, accounting for about 0.2% of genital tract malignancies. It often invades the myometrium, with focal, worm-like, or diffuse multinodular infiltration (4).

The latest WHO classification divides LGESS, HGESS, and undifferentiated uterine sarcoma (5). LGESS lacks specific clinical symptoms and is usually manifested as abnormal uterine bleeding, dysmenorrhea, and pelvic pain (6, 7). Therefore, it is often misdiagnosed as uterine myoma or adenomyosis. Its growth is slow, with a good prognosis, but it has a high recurrence rate and potential for metastasis. Pulmonary metastasis is uncommon but a common distant organ.

Clinically, it usually occurs in premenopausal women, with an average age of 42 to 53 years, and rarely in young women. Clinically, it can be manifested as respiratory symptoms such as dyspnea, chest tightness, chest pain, and cough. However, about 50% of patients have no pulmonary symptoms. Due to the lack of specificity of pulmonary symptoms, clinical misdiagnosis and missed diagnosis are common.

Radiologically, it is usually manifested as multiple or single nodules or masses, with a few patients having cystic cavities and cavitations (8), and is often accompanied by emphysema. When there are no obvious pulmonary symptoms, imaging can indicate the presence of lesions, reducing the rate of missed diagnosis and enabling timely diagnosis and treatment.

In terms of imaging, whole-body imaging is crucial for detecting atypical symptoms of ESS, such as bone pain. Although bone metastasis is rare, muscle and bone pain should raise suspicion of tumor metastasis, thus whole-body imaging can prevent the omission of rare metastatic sites, such as the ischium (9). In molecular imaging, particularly in PET/CT, it plays a significant role in assessing the recurrence and metastasis of endometrial cancer (10). FDG-PET/CT has excellent diagnostic performance in preoperative identification of lymph node metastasis and postoperative disease recurrence in endometrial cancer (11).

Macroscopically, it appears as single or multiple nodules in the lung parenchyma or subpleura, with clear boundaries, varying sizes, and a uniform gray-white or gray-brown cut surface with a fish flesh-like texture. In some cases, cystic cavities may be present. When nodules are large, liquefaction necrosis in the center can lead to cavity formation.

Microscopically, cells are arranged individually, in small clusters, or occasionally in sheets. Tumor cells resemble normal endometrial stromal cells, with indistinct cell boundaries, round or oval shape, uniform size, mild nuclear atypia, smooth nuclear membranes, scant or moderate cytoplasm, and fine chromatin. Structurally, tumor cells are densely packed, with round, oval, or short spindle shapes. They differentiate into endometrial stromal cells, surrounding thick-walled small vessels in a spiral pattern. There is little nuclear atypia, no tumor necrosis, and fewer than 5 mitoses per 10 HPF. Foam cells and inflammatory cells are present between tumor cells, with areas of fibrosis, hyalinization, or lymphangiomatosis. Morphologically, it is diverse, with possible smooth muscle differentiation, fibromyxoid change, sex cord-like differentiation, and endometrioid glands.

Immunophenotypically, CD10 and Vimentin are almost always diffusely and strongly positive, while ER and PR are often diffusely positive. CD10 is a relatively reliable marker, though sensitive but not specific, as cellular leiomyomas are also often CD10-positive. SMA, desmin, h-caldesmon, and S-100 are used in combination for diagnosis, especially when smooth muscle differentiation is present.

Molecularly, most cases have a t(7;17)(p21;q15)translocation, causing JAZF1 and SUZ12 fusion. Some cases show PHF1-JAZF1, EPC1-PHF1, or MEAF6-PHF1 rearrangements (12). Although molecular testing was not performed in this case, JAZF1 related gene fusion analysis can provide strong support for the diagnosis of low-grade stromal sarcoma in cases of atypical diagnosis or difficult differential diagnosis.

Given the rarity of pulmonary metastasis of low-grade endometrial stromal sarcoma, it is essential to differentiate it from the following tumors in pathological diagnosis:

1. Pulmonary leiomyoma can be distinguished by the absence of characteristic spiral arterioles in pulmonary metastasis of LGESS, despite possible smooth muscle differentiation. Immunohistochemical markers such as CD10, ER, PR, SMA, and desmin are helpful.
2. Pulmonary metastasis of HGESS is high-grade, with significant cellular atypia, more than 10 mitoses per 10 HPF, necrosis, and negativity for CD10, ER, and PR but positivity for CyclinD1 and possibly CD117 (13). Molecularly, it shows YWHAE-NUTM2 fusion.
3. Pulmonary metastasis of leiomyosarcoma is more common in uterine sarcomas, with longer tumor cells, significant atypia, increased mitoses, and necrosis. It expresses smooth muscle markers but is negative for CD10, ER, and PR.
4. Inflammatory myofibroblastic tumor has more inflammatory cells, spindle-shaped tumor cells without round or oval cells, and possible ALK positivity.
5. Metastatic carcinoma expresses epithelial markers like CK and EMA but not sarcoma markers. Some sarcomas may express CK, but LGESS is usually CK-negative and positive for CD10, ER, and PR.
6. Lymphohematopoietic tumors express lymphoid markers like LCA, CD20, CD3, and sometimes CD10 in follicular lymphoma but are negative for ER and PR.

In terms of treatment, the primary approach for most lung metastases of LGESS is surgical resection followed by±-hormone therapy. If the lesion is resectable, surgery is the preferred option. Hormone therapy should be selected based on the ER/PR status; it is not typically the first choice unless the lesion is inoperable or symptoms are severe. Radiotherapy is used for local control, while chemotherapy is reserved for advanced cases where hormone resistance or rapid disease progression has occurred.

In terms of prognosis, LGESS is characterized by slow growth, good prognosis, and high risk of recurrence and metastasis. Tumor emboli in the primary lesion indicate a high risk of recurrence and metastasis.

Pulmonary metastasis of LGESS is rare, the two cases in this paper had pulmonary metastasis 2 months and 11 years after surgery, indicating that metastasis can occur regardless of time. Therefore, more data are needed to study the diagnosis and treatment of pulmonary metastasis of LGESS and whether it can be prevented.

## Conclusion

The overall survival of LGESS is good, but the risk of long-term recurrence and metastasis is high, especially in patients with local or non-metastatic disease (14). Given the extreme rarity and diverse manifestations of pulmonary metastasis of LGESS, it is often confused with other tumors and misdiagnosed. Pathologists should integrate clinical history, imaging, histomorphology, and immunohistochemical markers when encountering pulmonary lesions, and review the primary lesion’s pathology to avoid misdiagnosis. Given the limited data on this disease, diagnosis and treatment are highly challenging. Reporting such cases can enhance clinical and pathologists’ understanding of the disease and help patients receive the best treatment.

## Data Availability

The original contributions presented in the study are included in the article/Supplementary Material. Further inquiries can be directed to the corresponding author.
